# Hypothesis: Cryptochromes and Brown Fat are Essential for Adaptation and Affect Mood and Mood-Related Behaviors

**DOI:** 10.3389/fneur.2012.00157

**Published:** 2012-11-05

**Authors:** Timo Partonen

**Affiliations:** ^1^Department of Mental Health and Substance Abuse Services, National Institute for Health and WelfareHelsinki, Finland; ^2^Department of Psychiatry, University of HelsinkiHelsinki, Finland

**Keywords:** basic rest-activity cycle, brown fat, clock, diurnal, evolution, natural selection

## Abstract

Solar radiation and ambient temperature have acted as selective physical forces among populations and thereby guided species distributions in the globe. Circadian clocks are universal and evolve when subjected to selection, and their properties contribute to variations in fitness within specific environments. Concerning humans, as compared to the remaining, the “evening owls” have a greater deviation from the 24 h cycle, are under a greater pressure to circadian desynchrony and more prone to a cluster of health hazards with the increased mortality. Because of their position in the hierarchy and repressive actions, cryptochromes are the key components of the feedback loops on which circadian clocks are built. Based on the evidence a new hypothesis is formulated in which brown adipocytes with their cryptochromes are responsive to a broad range of physical stimuli from the habitat and through their activity ensure adaptation of the individual. The over-activated brown adipose tissue with deficient cryptochromes might induce disrupted thermoregulation and circadian desynchrony, and thereby contribute to lowered mood and pronounced depressive behaviors.

## Introduction

Darwin (1859) concluded that “*each at some period of its life, during some season of the year, during each generation or at intervals, has to struggle for life, and to suffer great destruction. When we reflect on this struggle, we may console ourselves with the full belief, that the war of nature is not incessant, that no fear is felt, that death is generally prompt, and that the vigorous, the healthy, and the happy survive and multiply*.” In this article, I follow the ideas of natural selection and the struggle for life and study the note of “*the vigorous, the healthy, and the happy*” to present a new hypothesis.

A view that solar radiation and ambient temperature have acted as selective forces among human populations and thereby guided species distributions has gained population genetic support. The genetic basis for adaptation to the climate-mediated selection was elucidated in a recent scan of the human genome using 61 worldwide populations (Hancock et al., 2011). Its analysis of the biological pathways that have been targeted by natural selection indicated two sets of genes: genes that are keys to the differentiation of brown adipocytes, and those whose regulation makes a difference in response to ultraviolet radiation.

Concerning genetic variants, among the best signals for adaptation to the climate-mediated selection were those in genes that have a role in heat and cold tolerance. In particular, a common genetic variant (an amino-acid-changing single-nucleotide polymorphism) in *TRIP6* was associated with absolute latitude, and with minimum winter temperature (Hancock et al., 2011). This gene is known to interact with proteins that regulate resting metabolic rate, and the thyroid-regulated energy balance.

These findings suggest that brown adipocytes and circadian clocks, of whose components the cryptochromes do react to ultraviolet radiation (Foley et al., 2011), are not only relevant to survival and adaptation, but also targeted by natural selection. Herein, I hypothesize that circadian clocks in brown adipocytes are relevant to mammalian adaptation. Further, the cryptochromes are of key importance here, because of their evolutionary roots and current position in the hierarchy of the components of circadian clocks. They favor the odds for survival from hazards caused by circadian desynchrony and the over-activated brown adipose tissue.

From the point of view above, to start with, I shall discuss about a role of circadian clocks.

## Circadian Clocks

Circadian clocks are universal (Dvornyk et al., 2003; Hut and Beersma, 2011) and evolve their properties when subjected to selection (Kannan et al., 2012). Species have developed these mechanisms to synchronize their cellular oscillations to environmental cycles. Such synchrony helps in anticipation of routine fluctuations in environmental conditions and in adaptation to these changes. When life emerged 3.5–3.9 billion years ago, the length of day was about 14 h and ultraviolet radiation was not filtered by the atmosphere. So, in the beginning of life, circadian clocks met light-dark transitions with the approximate period of 14 h and were readily involved in protection from ultraviolet radiation. Thereafter, the period has lengthened and circadian clocks have adopted periods longer than 14 h up to the current one of 24 h as the reference. New properties were needed when the routine change of the seasons, with the routine variations in photoperiod and geomagnetic activity, started to challenge the functions of circadian clocks among those species that were living in locations further away from the equator. It may have evolved the properties of temperature compensation for circadian clocks in animals and non-shivering thermogenesis for mammals.

Hence, circadian clocks are built on properties generating (metabolic) oscillations within the ultradian (i.e., shorter than 24 h) range, having the primary task for protection from ultraviolet radiation. Of the circadian clock components, the cryptochromes evolved from the family of DNA photolyase proteins, implying that control of the light-induced effects and protection from ultraviolet radiation share a common evolutionary origin (Heijde et al., 2010). Cryptochromes react not only to ultraviolet radiation (Foley et al., 2011), but also to light exposure (Hirayama et al., 2009; Foley et al., 2011) and to geomagnetic activity (Gegear et al., 2010; Foley et al., 2011), and take part in regulation of the thyroid-regulated energy balance involving brown adipose tissue in mammals (López et al., 2010). Therefore, a pacemaker generating the ultradian rhythms might be the primary one for the body, as it is the oscillator with a shorter period which sets the pace for oscillators with longer periods and not vice versa.

### Circadian clock read-outs are relevant to adaptation

Natural variation of the circadian clocks can contribute to variations in fitness within specific environments. With a circadian period being well matched to the environment, plants contain more chlorophyll, fix more carbon, grow faster, and survive better than plants with circadian periods being more different from their environment and gain competitive advantage (Dodd et al., 2005). The degree of a deviation of the internal circadian period from the external 24 h cycle in nature is inversely related to the lifespan in many rodent and primate species (Wyse et al., 2010), and this mismatch of the circadian period with the environmental schedules have adverse effects on health in humans (Zheng and Sehgal, 2012).

Concerning humans, African American individuals have the free-running circadian period of 24 h 10 min on average, whereas Caucasian American individuals have that of 24 h 22 min on average (Eastman et al., 2012), demonstrating an increasing gradient in the innate circadian period and its variability away from the equator. However, the benefit from a period being slightly longer than 24 h (Bagheri et al., 2008) is easily lost due to lengthening of the intrinsic circadian period furthermore on average by 12 min in May and by 16 min in June in the northern hemisphere (Smith et al., 2009). The benefit is also reduced by deprived sleep (Burgess, 2010) that is common at higher latitudes particularly during summer and winter (Ohayon and Partinen, 2002; Bratlid and Wahlund, 2003), and this loss favors the emergence of circadian desynchrony, i.e., the condition where the intrinsic circadian clocks become misaligned with the daily photoperiodic cycles.

Normally, natural environmental light and ambient temperature of the external 24 h cycle act together to dictate the phase and to entrain circadian clocks (Boothroyd et al., 2007), but the change of seasons tends to challenge their functions, and therefore, for example, in plants the lengthened period serves to delay the onset of flowering until later in the season, and it could prove advantageous in avoiding the late-spring light but cold weather (Michael et al., 2003). Since most biochemical reactions respond robustly to temperature, the evolution of mechanisms to buffer the effects of day-to-day changes in ambient temperature is expected to favor adaptation (François et al., 2012).

Pittendrigh and Takamura (1987) discovered that change in the level of response to all night-length measurements (durations of nighttime darkness, rather than those of day-length) is a fundamental property of the circadian clock, and that adjustment to temperature and latitude are not separate: at each new latitude invaded by fruit flies, natural selection retunes the temperature dependence of photoperiodic responses to assure that the individual’s reproductive activity will exploit the same temperature range to which the rest of its physiology has adapted at earlier latitudes (Pittendrigh, 1993). It is of note, concerning day-active animals here, that transcription of the cryptochrome genes is induced in the evening (Lincoln et al., 2002), and that the heat-induced phase shifts of the circadian clock are severely reduced in the cryptochrome loss-of-function mutants (Kaushik et al., 2007).

### Diurnal “owls” are not easily aligned nor adapted

Recently, a hypothesis has been presented, according to which circadian desynchrony that has accompanied the availability and uninterrupted use of electric lighting in the modern world induces metabolic dysfunction predisposing to obesity (Wyse et al., 2011). This hypothesis is supported by data demonstrating that there is a shortening of workday sleep together with a delay in average chronotype as the date of data collection moves forward (Roenneberg et al., 2012). These data suggest that there is a decreasing strength of time-givers at societal level due to not only spending more time working indoors and less time outdoors during the day, but also receiving more light pollution during the night. Moreover, it is likely that there are additional reasons for the progressive delay in average chronotype and the hypothesized predisposition to obesity, such as less exposure to cold during the winter, less restricted meals, or more sedentary activities in the evening, e.g., with drinking alcohol and smoking, all of which might link to the activity of brown adipose tissue (for review, see Tan et al., 2011).

If this holds true, then why there are more morning-types among the elderly? I see that this is due to the cross-sectional study design with which data have been collected. Currently, there are no longitudinal data on the same individuals assessed with the same method available, except from a cohort study in which 190 twins were followed-up for 5 years (Koskenvuo et al., 2007). These preliminary results yielded that in 63% of the individuals the chronotype remained the same and in 19% similar, but 7% changed from evening-types to morning-types and 11% from morning-types to evening-types. As such this finding disagrees with the common view according to which persons become more of morning-types with aging.

An explanation may be, and I call it here as the diurnal owl hypothesis, that evening-type of persons (“evening owls”) tend to die younger than morning-type of persons (“morning larks”), but whether this is because of a drive for delays in chronotype and subsequent circadian desynchrony, it is not known and needs verification. However, this view might not be far-fetched, since thus far all the health hazards assessed appear to cluster and be more frequent among the evening-types, including unhealthy dietary habits (Kanerva et al., 2012), smoking and nicotine dependence (Broms et al., 2012), and the increased odds for depressive, anxiety, and substance use disorders (Reid et al., 2012), insomnia (Merikanto et al., 2012a), hypertension, and type 2 diabetes (Merikanto et al., 2012b). Evening-types by definition do have greater deviations of their circadian period from the 24 h cycle on average than others (Duffy et al., 2001), but it is not currently known in detail how this property might link to a greater morbidity or mortality in humans.

Next, I shall discuss about a role of brown adipocytes.

## Brown Fat

I see that there is currently enough evidence to claim that “*the vigorous, the healthy, and the happy*” have the aligned circadian clock. In mammals, brown fat has a key role in heat and cold tolerance, since in its activated state it generates heat, a phenomenon called as non-shivering thermogenesis. It is currently evident that brown adipose tissue is a highly active tissue, rather than only “*a form of embryonic adipose tissue*” (Sheldon, 1924) or the so-called hibernating gland, and it is highly prevalent in adult humans (Lee et al., 2011). It can be activated in nearly all but its activity is reduced in those who are overweight or obese (van Marken Lichtenbelt et al., 2009; for review, see Enerbäck, 2010).

Concerning mammals, another view to keep in mind is the conclusion by Kleitman (1949) saying that “*a physiological rhythm, specifically the diurnal rhythm, is essentially a metabolic cycle*.” I want to contribute to this view, extend it and hypothesize that there is a link between mood and the basic rest-activity cycle (Kleitman, 1961), which is not equal to the behavioral rest-activity rhythm but an ultradian physiological rhythm. The basic rest-activity rhythm is closely linked to the activity of brown adipose tissue (Blessing et al., 2012). Here, I hypothesize that a change in the activity of brown adipose tissue has an effect on mood, and that the over-activated brown adipose tissue lowers mood.

### Heat generated by brown fat is central to all oscillations

Thus far, there is preliminary evidence demonstrating that brown adipose tissue was clearly over-activated in two suicide cases with depressive disorder (Huttunen and Kortelainen, 1990), but on the basis of these data it cannot be judged whether the finding was specific. However, it provides a basis for a view that the activation of brown adipose tissue improves cold tolerance at the cost of heat tolerance, triggering anxiety, and psychomotor agitation, and in the end affects mood in a negative way during the late-spring and early summer (Hiltunen et al., 2011). To be specific I hypothesize that it is the compromised heat tolerance which triggers deaths from suicide during a particular time of the year and explains part of the variance in seasonal mortality peaks of suicide.

The circulating blood is a carrier of heat which follows not only the circadian rhythm of core body temperature guided by the suprachiasmatic nuclei (Buhr et al., 2010), but also the ultradian fluctuation in non-shivering thermogenesis generated by brown adipose tissue (Ootsuka et al., 2009). Furthermore, there is an ultradian rhythm of e.g., free corticosterone in the blood (Qian et al., 2012; Waite et al., 2012) that translates into synchronized rhythms of free glucocorticoid hormone in peripheral (the subcutaneous tissue) and central (the hippocampus) tissues (Qian et al., 2012). Ultradian rhythms are superimposed on the circadian rhythms, and under conditions where the circadian rhythms damp out, the ultradian rhythms remain (Eastman and Rechtschaffen, 1983).

The resistance of ultradian rhythms to interventions which lengthen the period of free-running circadian rhythms, especially that of nocturnal ultradian rhythms (Prendergast and Zucker, 2012), contrasts with the slowing of other biological timing processes, suggesting unique control mechanisms for generation of ultradian and circadian rhythms (Prendergast et al., 2012). There is evidence that brown adipose tissue may be a site of interaction between metabolic and circadian systems (van der Veen et al., 2012). It appears that a non-transcriptional pathway for the metabolic cycle engages the circadian clock, thereby enhancing clock performance (van Ooijen and Millar, 2012). Changes in the cellular metabolic state could be the cause, not just the result, of neuronal activity (Wang et al., 2012), and if this holds true, then the ultradian rhythms in brown adipose tissue may lead neuronal activity. Importantly, in support to this view, changes in the activity of brown adipose tissue can guide the overall maintenance of the circadian rhythm of core body temperature (Yang et al., 2011).

### Once being activated brown fat is not easily quenched

Brown adipose tissue may in fact be an active pacemaker tissue, having activity in a range of ultradian (Ootsuka et al., 2009) to infradian (Zukotynski et al., 2010) oscillations. Here, I hypothesize that it is so, because (a) in brown adipose tissue, there is abundance of peroxisomes that are responsive to thermogenic stimuli (Bagattin et al., 2010), (b) when brown adipose tissue is activated by cold in humans, glucose uptake increases by 12-fold, accompanied by a twofold increase in perfusion, whereas in response to insulin there is high glucose uptake without increased perfusion (Orava et al., 2011), and (c) the increased glycolytic capacity of brown adipose tissue involves a twofold increase in the activity of phosphofructokinase (Cooney and Newsholme, 1982).

Brown adipose tissue assists in adaptation to cold challenges through futile substrate-energy cycles. One such cycle is the futile cycle, burning up ATP molecules between fructose-1,6-diphosphatase and phosphofructokinase activities, that generates a metabolic signal having a circadian rhythm (Zhang et al., 2006). In addition, peroxiredoxins being abundant in brown adipose tissue contribute to the seasonal behavior under conditions in which the coordination by the suprachiasmatic nuclei is weak (Epperson et al., 2004), being reminiscent of those in which the time-keeping mechanisms are challenged (Wagner et al., 2008) and circadian desynchrony is likely to emerge (van Oort et al., 2007).

Once brown adipose tissue has become metabolically active, it is more resistant to becoming quiescent (Zukotynski et al., 2010) and, as I hypothesize, it is then more subject to defects in heat tolerance. It is of note here that non-thermal stimuli, such as the photoperiod, may also affect the development of brown adipose tissue, and that at thermoneutral conditions the inhibitory action of a long photoperiod on the activity of brown adipose tissue is lost only during the early summer (Heldmaier et al., 1981), indicating this period of the year to be permissive to over-activation of brown adipose tissue. Mismatch emerging from conflict between signals of the photoperiod and those of ambient temperature (i.e., long and sunny but still cold days at high latitudes during the late-spring) might have relevance among those suffering from depressive episodes (Huttunen and Kortelainen, 1990; Partonen et al., 2004; Hiltunen et al., 2011).

Next, in order to converge, I shall discuss about a role of cryptochromes.

## Cryptochromes

I see that there is currently some evidence to suggest that “*the vigorous, the healthy, and the happy*” also have brown adipose tissue which is not easily over-activated. The proteins encoded by the cryptochrome genes (Hsu et al., 1996) are likely to be of key importance here, because of their unique role in the core of circadian transcriptional feedback loops (Hirota et al., 2012; Padmanabhan et al., 2012), via their interaction with 5′-AMP-activated protein kinase (Um et al., 2011; for review, see Jordan and Lamia, 2012) whose activity in turn controls the activity of brown adipocytes (López et al., 2010). In addition, there is a crossroad of actions at glucocorticoid receptors that provides a link between brown adipocytes and cryptochromes. On the one hand, a nuclear isoform of TRIP6 increases glucocorticoid-receptor-mediated transcription, and it is required for the transrepression of glucocorticoid receptor by NF-κB (Diefenbacher et al., 2010). On the other hand, cryptochromes directly take part in glucocorticoid-receptor-mediated repression of glucocorticoid synthesis (Lamia et al., 2011), and in blockade of the NF-κB signaling pathway (Narasimamurthy et al., 2012).

I hypothesize that the CRY1 and CRY2 proteins play this key role in brown adipose tissue, similar to their role in other tissues analyzed so far, where CRY1 (and possibly CRY2 as well) after the encoding gene being induced by melatonin (Hazlerigg et al., 2004) might mediate the inhibitory effect of melatonin on cyclic adenosine monophosphate reactions in brown adipocytes. This effect can be achieved directly through interaction of the CRY1 and CRY2 proteins with the G_s_α subunit of heterotrimeric G protein (Zhang et al., 2010) or that of CRY1 (and possibly CRY2 as well) with adenylyl cyclase (Narasimamurthy et al., 2012). It reduces accumulation of cyclic adenosine monophosphate in response to G protein-coupled receptor activation and leads to inhibition of downstream reactions (Zambon et al., 2005; O’Neill et al., 2008).

Lack of this inhibition of downstream reactions in dopaminergic cells, at least in the striatum (Park et al., 2005) but potentially elsewhere as well such as in the ventral tegmental area (Gonzalez and Aston-Jones, 2008; Hampp et al., 2008; for review, see Borrelli et al., 2008), results in increased depression-like behaviors. Under the short photoperiod the melatonin signal is not sufficient to inhibit the cyclic adenosine monophosphate response during the second half of the night (MacGregor and Lincoln, 2008), but adenosine elicits through the melatonin-sensitized adenosine A_2b_ receptor an increase in the transcription of cyclic adenosine monophosphate-inducible genes (von Gall et al., 2002), which characterizes the so-called sleep-sensitive circadian period of the individual. Being abnormally activated, or over-activated, this cascade provides a key to the antidepressant action of sleep deprivation protocols in the depressed (Wirz-Justice and Terman, 2012).

### Cryptochromes might guide brown fat activity

Here, the CRY1 protein may however be second to the CRY2 protein (Dardente et al., 2007; Wongchitrat et al., 2009; Padmanabhan et al., 2012). This argument is supported by findings from knock-out mice which demonstrate that without the latter one the circadian period is lengthened similar to “evening owls,” whereas without the former one it is shortened similar to “morning larks” (van der Horst et al., 1999; Vitaterna et al., 1999). It is strengthened further by those which show that the *CRY2* gene expression is abnormal when inbred-strain mice with the intrinsic level of high anxiety are deprived of sleep (Wisor et al., 2008), and when humans with bipolar type 1 disorder do remain depressed after the antidepressant sleep deprivation (Lavebratt et al., 2010).

If both CRY1 and CRY2 feedback loops are intact, the nuclear ratio of CRY1 to CRY2 proteins controls the period of the circadian clock, more CRY1 causing longer periods and more CRY2 causing shorter periods (Hirota et al., 2012). Similarly, data on the core clock gene encoded proteins (for review, see Ko and Takahashi, 2006) suggest and I speculate here that the nuclear ratio of RORA to RORB may control the period of the circadian clock, more RORA causing longer periods and more RORB leading to shorter periods (for review, see Partonen, 2012). However, there are gaps of knowledge, as the impact of melatonin on transcription of *CRY1* (Hazlerigg et al., 2004; MacGregor and Lincoln, 2008), the roles of *PER2* and *CRY1* as a systematically driven genes that bear a signal of time in a correct phase throughout the tissues (Kornmann et al., 2007; Hughes et al., 2012), and the cryptochrome-independent influence of the PER2 protein on transcription of the *ARNTL* (*BMAL1*) gene (Schmutz et al., 2010; Ye et al., 2011) are not yet understood in depth.

The nuclear ratio of CRY1 to CRY2 proteins (Hirota et al., 2012) may be of key importance here, as it bears an (inhibitory) effect on the functions of the G_s_α subunit of heterotrimeric G proteins (Zhang et al., 2010). If it does, then a sustained change in this ratio may impair the functions of the dopamine D2 receptors in the retina. It may affect adaptation to light and compromise the DRD2-mediated signaling in the control of circadian clocks (Yujnovsky et al., 2006), and release the inhibition of non-shivering thermogenesis in brown adipose tissue (Ootsuka et al., 2007). Such release might underlie some key features of the phenotype in depressed individuals, e.g., defects in thermoregulation, blunted circadian amplitudes, and elevated core body temperatures during the night, a marked loss of weight, and abnormalities in the theta oscillation during wakefulness and during sleep. It is not known, whether these phenotypic characteristics, or some of them if any, are due to the over-activated brown adipose tissue in the depressed.

However, a question remains here: why is the activity of brown adipose tissue not inhibited by the functions of dopamine D2 receptors? I see that this could be a mixed result from, e.g., the increased antagonism of dopamine D2 receptors (medication blocking these receptors), the up-regulated coupling between dopamine D1 and D2 receptors (Pei et al., 2010), or the relative shortage of light exposure (dark fall and winter mornings, time spent indoors during the day, or photophobic behaviors) that are known to coincide with depressive symptoms.

Currently, it is not known, whether the knock-out or the knock-down of the *CRY2* gene in the whole organism or specific to a tissue produces any change in the activity of brown adipose tissue or in anxiety-like or depressive-like behaviors. Furthermore, it is not yet known how mutated CRY2 proteins (McCarthy et al., 2009) influence. It is of note here that the human CRY2 protein has two isoforms produced by alternative splicing, while the human CRY1 protein has none. This may be relevant if one takes a scenario where there is a link between the circadian clock to cold tolerance (or heat tolerance) and alternative splicing is used to fine-tune the individual’s responses to the environment, similar to that in fruit flies (Majercak et al., 1999) or in plants (Seo et al., 2012). To the end I see that there are not yet enough data to conclude, but intuitively it seems essential, that “*the vigorous, the healthy, and the happy*” have fully functional cryptochromes.

Finally, I shall present my hypothesis more in details.

## Hypothesis

Current knowledge gaps include the lack of data on the effect of cryptochromes on complex behaviors such as anxiety and depressive-like behaviors. In addition, there is only indirect evidence for the link between the activity of brown adipose tissue and the level of anxiety. So far, this link has been observed in mice with the knock-out of the histidine decarboxylase gene. These mice have the increased amount of brown adipose tissue (Fülöp et al., 2003) and display the increased level of anxiety in behavioral test settings (Acevedo et al., 2006a,b). However, there are no direct data derived from the same set of experiments to demonstrate, whether the amount or the activity of brown adipose tissue correlates with the measures of anxiety in behavioral tests in these or any other knock-out mice nor inbred mouse strains differing by their baseline levels of anxiety.

To test the hypothesis, I have together with my colleagues planned a series of experiments (*CRY2 Expedition*) as follows.

### Mouse studies

On one hand, we have started the assessment of *Cry2* knock-out mice and anxiety-ranked inbred-strain mice. For assessment, we measure the intensity of anxiety and depressive-like behaviors, with a set of behavioral tests, and we scan the intensity of brown adipose tissue activity, with mouse positron emission tomography. These behavioral and physiological intensities are assessed both at baseline and after activation of brown adipose tissue.

What we expect to see as a result of these experiments with mice, it is the following: (1) the higher the level of anxiety or depressive-like behaviors of inbred mouse strains is, the higher the activity of brown adipose tissue is, (2) *Cry2* knock-out mice have a higher level of anxiety or depressive-like behaviors and a higher activity of brown adipose tissue than their wild-type controls, and (3) after activation of brown adipose tissue by cold exposure (or with drugs) there is an increase in the activity of brown adipose tissue in both *Cry2* knock-out mice and their wild-type controls, the increase being greater (corresponding to over-activation) in *Cry2* knock-out mice, and an increase in the anxiety or depressive-like behaviors in *Cry2* knock-out mice only. Experiments with brain-specific or brown-adipose-tissue-specific rescues of *Cry2* are thereafter needed to demonstrate the hierarchy between central and peripheral tissue oscillators, and to point out those mechanisms of action which translate the cellular activity into the anxiety or depressive-like behaviors.

### Human studies

On the other hand, studies with depressed patients are needed to translate the findings into humans. We are planning a set of studies where after the clinical assessment of diagnoses we scan depressed patients using human positron emission tomography both at baseline and after activation of brown adipose tissue by cold exposure. In addition, we are planning another set of studies on suicide victims with depressive disorder, whom we assess with autopsy, having our focus on the reciprocal projections between the brain and brown adipose tissue.

With these studies we want to (a) replicate the findings of over-activated brown adipose tissue in suicide victims with depressive disorder as compared with their controls, (b) measure the *CRY2* expression levels and determine the amount of CRY2 protein in a nuclear fraction vs. a cytosolic fraction of cells in the brain regions of our interest and in brown adipocytes among suicide victims with depressive disorder as compared with their controls, and (c) elucidate the questions whether “evening owls” or those with a current depressive episode have a higher activity of brown adipose tissue than their controls, and whether brown adipose tissue becomes easily over-activated by cold exposure in “evening owls” or those with a current depressive episode only.

### A new hypothesis

**Figure 1:**
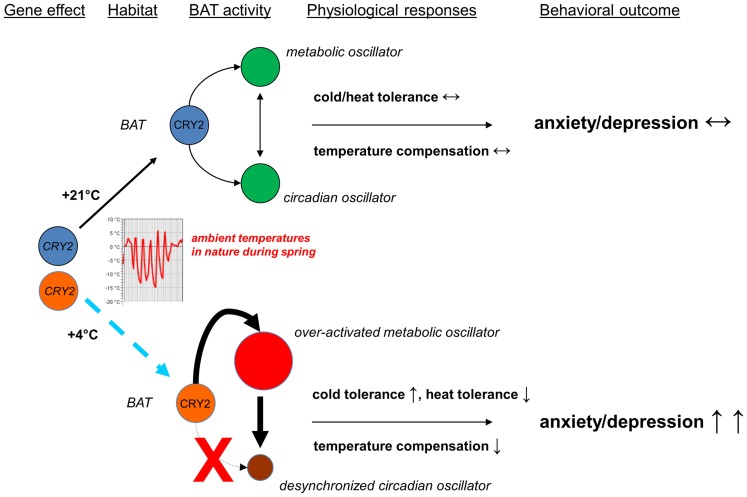
**Scheme of the hypothesis integrating the circadian control, brown adipose tissue activity, and mood**. Rapid changes in ambient temperature (cold nights, warm days) which are typical at temperate latitudes during the late-spring activate brown adipose tissue, once being activated it will not cool down easily and remains therefore prone to over-activation. Because of a genetic effect (*CRY2* gene mutations or common variants which compromise the function of CRY2 proteins) or abnormalities in the post-translational control of CRY2, brown adipose tissue is over-activated easily. It is currently not known, whether this kind of over-activation of brown adipose tissue, hypothesized to lead to the increased cold tolerance but to cause defect in tolerance to heat, characterizes mood disorders and anxiety disorders, and whether it contributes to the seasonal peaks in their occurrence and mortality rates for deaths from suicide, but it needs to be tested against experiments and observations.

Here, I formulate my hypothesis (see Figure 1) to constitute the two predictions which are being tested against experiments and observations as follows.

Prediction #1: Cryptochrome (especially cryptochrome 2) functions are essential for normal mood. If these are compromised, control of the activity of brown adipocytes is lost, leading to the over-activated brown adipose tissue.

Prediction #2: Brown adipose tissue activity regulates mood and mood-related behaviors. The over-activated brown adipocytes generate heat, which, if sustained, disorders the pacemaker function and causes depressive-like behaviors or a depressed episode.

The rationale and the line of reasoning are based on the data which I have presented above and summarize as follows. Cryptochromes are, because of their position in the hierarchy and repressive actions, the key components of the core of the transcription-translation feedback loops on which circadian clocks are built. Brown adipocytes with their cryptochromes are responsive to the continuous flow of stimuli from the habitat, e.g., the photoperiod, ambient temperature, and geomagnetic activity, they “embank” these stimuli in the body, and with their activity they guide the ultradian rhythms and thereby contribute to mood and mood-related behaviors.

## Conflict of Interest Statement

The author declares that the research was conducted in the absence of any commercial or financial relationships that could be construed as a potential conflict of interest.
